# Supplementary material for: Novel Non-Cyclooxygenase Inhibitory Derivative of Sulindac Inhibits Breast Cancer Cell Growth In Vitro and Reduces Mammary Tumorigenesis in Rats
Source: Cancers (Basel). 2023 Jan 20;15(3):646. doi: 10.3390/cancers15030646 (PMC9913705; doi:10.3390/cancers15030646)
Supplement: Supplementary file 1 [file cancers-15-00646-s001.zip › cancers-2121305-supplementary.pdf]

p-VASP^{Ser239}

DMSO

Treatment Time

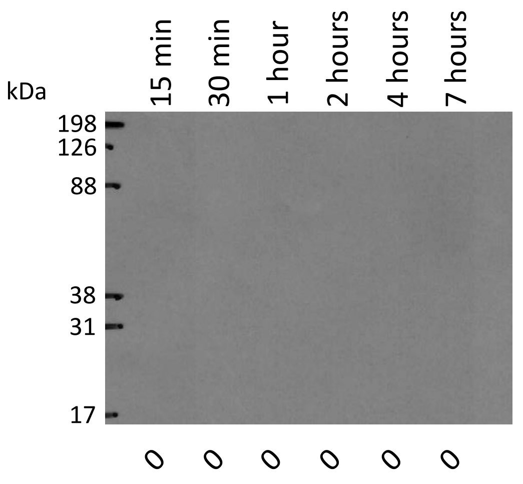

7.5 µM SSA

Treatment Time

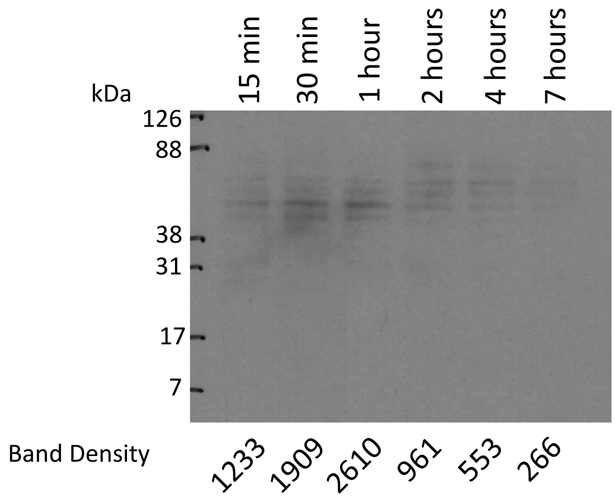

Cyclin D1

DMSO

Treatment Time

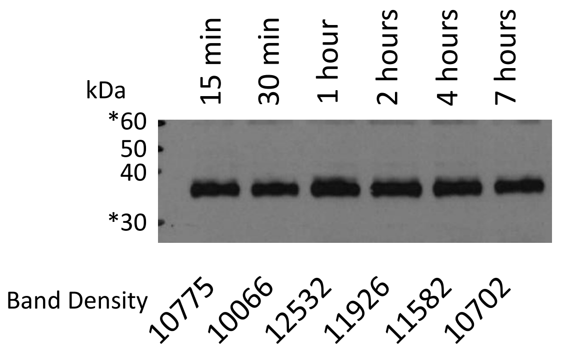

7.5 µM SSA

Treatment Time

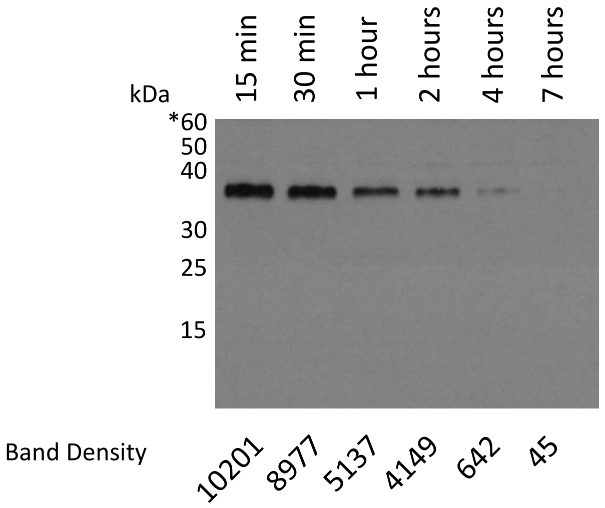

*To reduce error from separate gels/blots, blots were cut at indicated markers prior to probing with primary antibody.

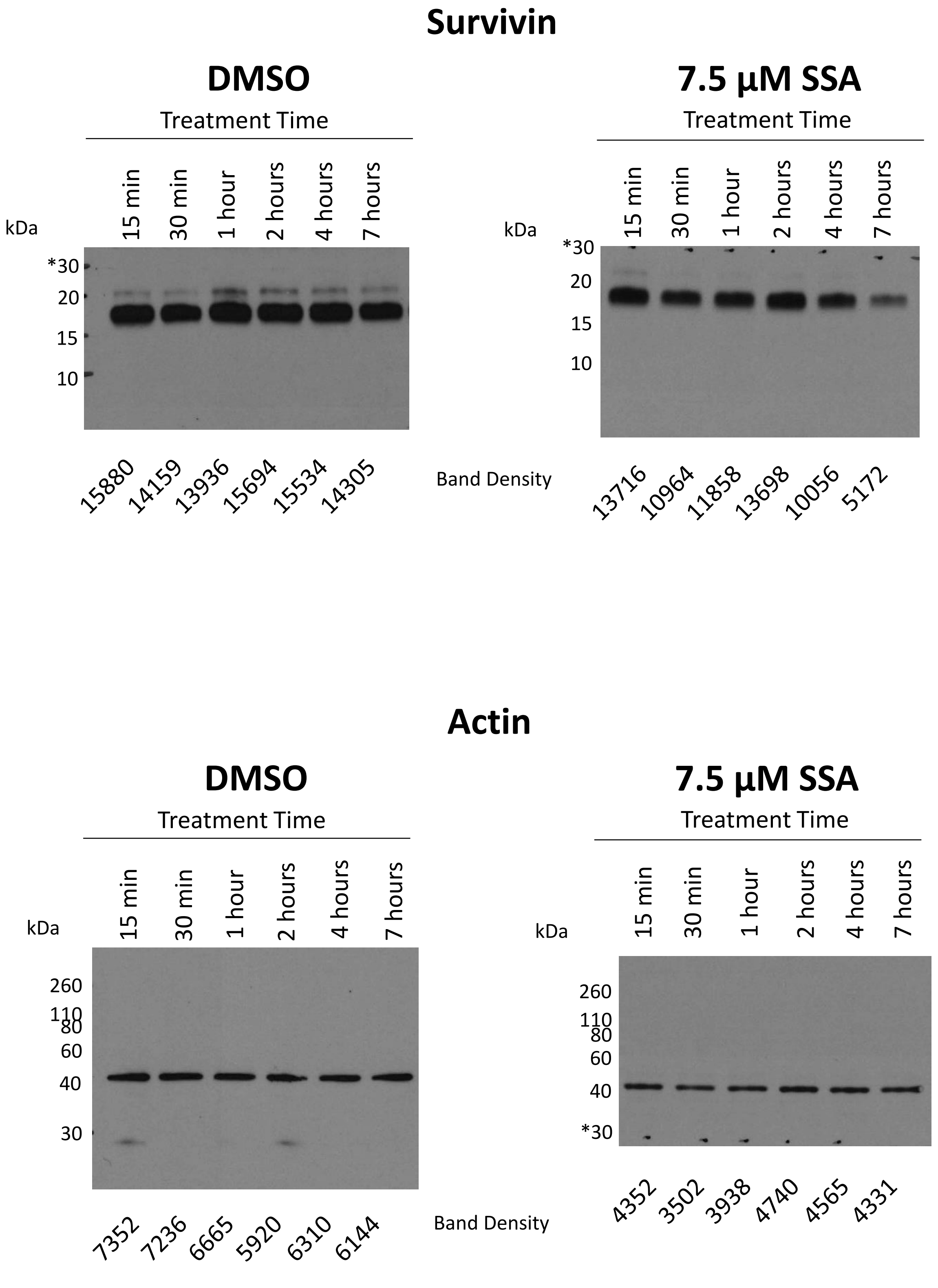

*To reduce error from separate gels/blots, blots were cut at indicated markers prior to probing with primary antibody.
